# Effect of temperature and pre-stretch on the dynamic performance of dielectric elastomer minimum energy structure

**DOI:** 10.1038/s41598-024-66566-0

**Published:** 2024-07-04

**Authors:** Zhipeng Wang, Qiaowei Xu, Yanmin Zhou, Gang Li, Bin He

**Affiliations:** 1 State Key Laboratory of Intelligent Autonomous Systems, Shanghai, 201109 China; 2 Frontiers Science Center for Intelligent Autonomous Systems, Shanghai, 201109 China; 3 College of Electronics and Information Engineering, Tongji University, Shanghai, 201804 China

**Keywords:** Dielectric elastomer (DE), Dielectric elastomer minimum energy structure (DEMES), Nonlinear dynamics, Pre-stretch effect, Soft robot, Engineering, Mathematics and computing

## Abstract

Dielectric Elastomer Minimum Energy Structures (DEMES) have the ability of actively adjusting their shape to accommodate complex scenarios, understanding the actuation mechanism of DEMES is essential for their effective design and control, which has rendered them a focus of research in the field of soft robotics. The actuation ability of DEMES is usually influenced by external conditions, among which the electromechanical properties of DE materials are highly sensitive to temperature changes, and the pre-stretch ratio of DE materials has a significant impact on the dynamic performance of DEMES. Therefore, it is necessary to study the effects of temperature and pre-stretch ratio on the nonlinear dynamic behavior of DEMES. In this paper, in response to the lack of research on the influence of DE pre-stretch ratio on the actuation characteristics of DEMES, this paper proposes a systematic modeling and analysis framework that comprehensively considers pre-stretch factors, temperature factors, and viscoelastic factors, and establishes the motion control equation of DEMES affected by the coupling effect of DE pre-stretch ratio and temperature. The proposed analytical framework is used to analyze the evolution of the electromechanical response of DEMES under voltage excitation under the coupling of DE pre-stretch ratio and temperature. The results indicate that the bending angle, inelastic deformation, resonant frequency, and dynamic stability of DEMES can be jointly adjusted by the DE pre-stretch ratio and ambient temperature. A low pre-stretch ratio of DE can lead to dynamic instability of DEMES, while appropriate temperature conditions and higher pre-stretch ratios can significantly improve the actuation ability of DEMES. This can provide theoretical guidance for the design and deformation control of DEMES.

## Introduction

Soft robots are currently significant topics of scientific frontier research. There are various forms of driving for soft robots, such as fluid drive^1–3^, magnetic drive^4^, SMA drive^5,6^, and chemical drive^7^. However, these driving methods have drawbacks in terms of complex production, slow response, and challenging control, resulting from material characteristics and structural design. Compared with other driving methods, electrically driven soft robots have the advantages of fast response and simple control^8–11^, gradually becoming a research hotspot in recent years.

Dielectric Elastomer Actuator (DEA) has the characteristics of biological inspiration and bionics, and their practicality and environmental adaptability play a crucial role in the rapid development of software actuators. In the field of soft actuators based on DE, there has been considerable research progress on the electromechanical and actuation characteristics of actuators during operation. However, research on external factors that may have periodic or stable effects on the electromechanical and actuation characteristics of soft actuators based on DE is not complete. It should be noted that soft actuators are currently applied in various environments, and each working environment has an undeniable impact on the actuation performance of DEAs. For example, changes in ambient temperature may weaken the load capacity of DE drives; The application of AC voltage at different frequencies can also lead to different working efficiency of DEAs. Therefore, it is very important to study the influencing factors of the actuation characteristics of soft actuators based on DE. The research results have certain reference significance for the design and application development of DEAs.

DEA is an electrically driven smart structure that consists of a pre-stretched dielectric elastomer membrane and two flexible electrodes^12–14^. Dielectric elastomer (DE) can be applied to the Dielectric Elastomers Minimum Energy Structure (DEMES). Khurana et al. provide a theoretical framework for investigating the non-linear dynamics of Smart Composite Elastomer-based Minimum Energy Structures (SCEMES)^15^, the efficient semi-analytical framework would be crucial in developing new actuators, smart devices and soft robots for a variety of advanced engineering and medical applications. Subramaniya Siva et al. present a finite element framework for simulating the quasi-static response of DEMES^16^, research results can find its potential use in designing the futuristic DEMES through topological optimization of the compliant electrode and frame geometry together with material anisotropy of the elastomer. Singh et al. employ a physics-based nonaffine material model proposed by Davidson and Goulbourne^17^, and the governing equation for dynamic motion is established using Euler–Lagrange’s equation of motion for conservative systems. Sharma et al. proposed a system development of a command forming scheme for controlling residual vibration in electrically driven planar DEA^18^, and the research results can provide reference for the design of DEAs open-loop control systems.

In addition, DE is a typical viscoelastic material, and viscoelastic relaxation and creep have a great influence on its electromechanical properties, making the deformation highly frequency-dependent^19–23^. Consequently, understanding the influence of viscoelasticity on the dynamic behavior of DEAs is crucial for the design of these soft robots^24–29^. Khurana et al. presents the dynamic modeling and analysis of a particle-reinforced and pre-stressed electro-magneto-viscoelastic plate actuator^30^, the viscoelastic factors of the membrane were fully considered to evaluate the stability, periodicity, jumping phenomenon, and resonance behavior of the actuator. Kumar et al. studied the effects of environmental humidity and membrane anisotropy on the dynamic behavior and introduction of instability phenomena of viscoelastic actuators^31^, and the results showed that the influence of viscoelastic factors is significant and cannot be ignored. Although some studies have involved the viscoelasticity effect on dynamical behavior of DEMES^32–44^, a comprehensive modeling research that considering ambient factors to understand the actuation mechanism is still lacking and necessary. Meanwhile, DE can also be applied to tunable phonon structures. Sharma et al. developed an effective gradient based topology optimization method to maximize the bandgap width of DE material^45^, which is also an application of tunable phonon structures. Alam et al. attempted to effectively damping the vibration of intelligent layered plates using active feedback control methods through the study of DE parameters^46^.

The pre-stretch ratio of DE has an important effect on its dynamic characteristics. He et al. studied the influence of pre-stretch on the bending deformation of DEAs^47^. Li et al. found that the pre-stretch can improve the stabilization of DE in several ways, such as by eliminating the pull-in instability, by generating electrostriction and by improving the breakdown strength^48^. Kofod et al. explored the direct effects of pre-stretch on the electromechanical coupling of DEAs by introducing an electrostatic driving model with pure shear condition^49^. Eder Goy et al. analyzed the dynamic pull-in instability of biaxial pre-stretched DEs under electric loading^42^. Hence, researchers have obtained some mechanisms for the effects of pre-stretch on the dynamic properties of DE. In the case of DEMES, the dynamic response is more complicated by the coupling of DE and flexible frame, which lacks the analysis of the effect of pre-stretch on its dynamic behavior.

The variations in environmental factors (e.g., temperature, humidity, magnetic field, etc.) cause a change in the stiffness of the electropolymer, which in turn affects the dynamics of DE and DEMES. For instance, the temperature can influence the dielectric properties and elastic modulus of DE^50,51^. Zhang et al. carried out an experimental investigation on the temperature effect on the electromechanical properties of DEs^50^, and the results demonstrate that the electromechanical deformation initially increases and subsequently decreases as the temperature rises. Liu et al. considered the influence of temperature on dielectric constant during the establishment of free energy function of DEs^51^. In our previous work, we analysed the effect of temperature on the dynamic performance of DEMES and obtained the mechanism of temperature modulation of its dynamic stability^52^. In summary, some work has investigated the effect of temperature on the dynamical properties of DEMES, there is a lack of a systematic modelling framework integrated with pre-stretch, which is important for the design, control and analysis of this class of actuators.

In this paper, the effect of pre-stretch and temperature coupling on the electromechanical properties of DEMES is investigated. A systematic modelling framework with integrated consideration of environmental factors, pre-stretch and viscoelasticity is established. The modulation mechanism of the pre-stretch ratio and temperature factor on the dynamic behavior is revealed. The results of the study can provide a reference for the optimal design and precise control of DEMES based actuators. The remainder of the paper is organized as follows. Section “Problem description and material model” presents the problem statement and the DEMES material model considering the effects of pre-stretch and temperature. Section “Dynamic governing equations” presents the nonlinear differential governing equations for the coupled multi-physics fields of DEMES based on the Euler–Lagrange method. Numerical simulations and analysis of results analyzing the effects of pre-stretch and temperature on the dynamic properties of DEMES are given in Sect. “Numerical simulation”. Finally, Sect. “Conclusion” summarizes the discussion and conclusions.

## Problem description and material model

**Figure 1:**
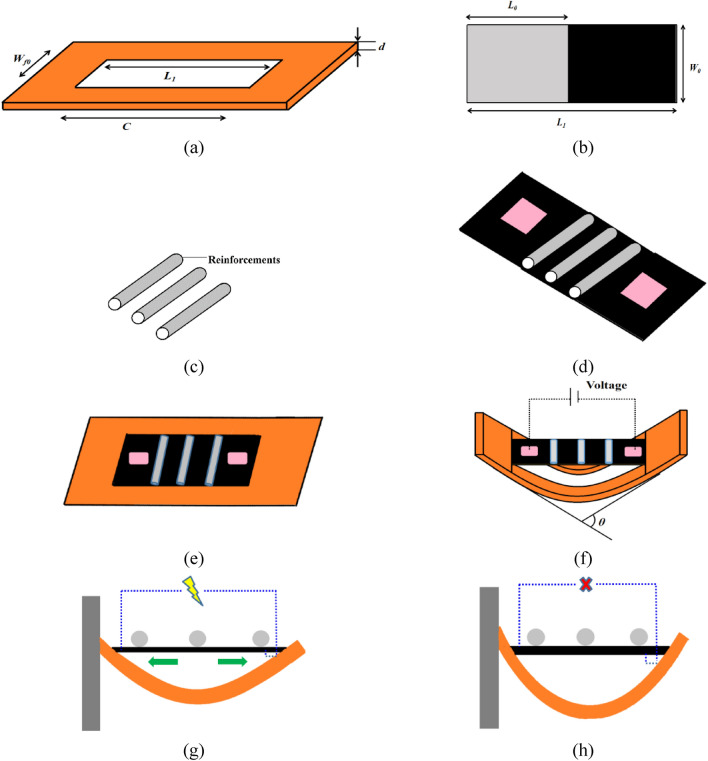
Schematic of DEMES (**a**) compliant frame, (**b**) DE membrane, (**c**) reinforcements (Anchoring fibrils), (**d**) assembly view of DE membrane, (**e**) assembly view of compliant frame, (**f**) equilibrium state, (**g**) no powered on state, and (**h**) power on state.

In order to analyze the effects of pre-stretch and temperature on the dynamic characteristics of DEMES, this section proposes an analytical model, the analysis model is derived from the author’s previous work on the influence of temperature on the dynamic performance of DEMES^52^. The schematic of the proposed DEMES is illustrated in Fig. 1. The main part of DEMES is a flexible frame with a center hole, as shown in Fig. 1a. The auxiliary part of DEMES is the pre-stretched DE membrane, which can generate pre-stretch stress, as shown in Fig. 1b. The holes in the flexible frame have a length *C*, a width *W*_*f*0_, and a thickness *d*. The DE membrane has a length of *L*_0_ and a width of *W*_0_ before stretch. When the DE membrane is under pure shear stress conditions (with a fixed width and only subjected to longitudinal stretch), its final length is *L*_1_, which is consistent with the length of the frame hole. Due to the setting of the pre-stretch condition in this paper as a pure shear mode, the DE membrane is laterally fixed with equally long fibers to prevent deformation, as shown in Fig. 1c and d. It is assumed that the DEMES is horizontal when the pre-stretched DE membrane is initially adhered to the flexible frame, as shown in Fig. 1e. The equilibrium state of DEMES under the action of pre-stretched DE membrane is shown in Fig. 1f. When the DE membrane adheres to the surface of the flexible frame, due to the pre-stretch stress inside the membrane, the flexible frame will deform and eventually reach its equilibrium state. When a voltage is applied, the DEMES deformation state is shown in Fig. 1g, which is in the released state. When no voltage is applied, the deformation state of DEMES is shown in Fig. 1h, which is in the contraction state under the pre-stretching effect of DE membrane. The next section will study the strain energy of DE membranes and the rheological model used to analyze DE membranes.

### Strain energy of DE membrane

**Figure 2:**
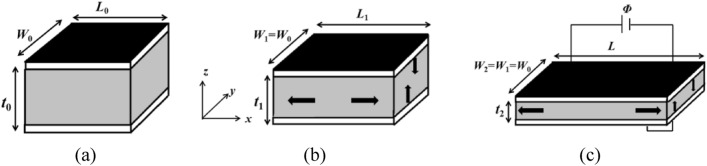
Schematic of a DE membrane in the (**a**) reference state, (**b**) pre-stretched state, and (**c**) actuated state.

A suitable pre-stretch ratio improves the actuation performance of the DE actuator. Figure 2 shows the deformation process of DE membrane subjected to pre-stretch and voltage loading^52^. The initial reference state of the DE membrane is shown in the Fig. 2a, with a length of *L*_0_, a width of *W*_0_, and a thickness of *t*_0_. After pre-stretching, the size of the DE membrane changes in various directions, which can be manifested as length expansion in the *x* direction and thickness contraction in the *z* direction. Due to the assumption of pure shear conditions, the width of the DE membrane in the *y* direction is constant, with a width of *W*_0_, as shown in Fig. 2b. When DE is subjected to an external voltage, the electrostatic stress causes further deformation of the membrane with a length of *L*, a thickness of *t*_2_, and a constant width of *W*_0_, as shown in Fig. 2c.

Based on the incompressibility assumption, the pre-stretch of DE membrane along three main directions (*x*, *y*, and *z*) can be expressed as:

1 $$\lambda_{P1} = \frac{{L_{1} }}{{L_{0} }},\;\lambda_{P2} = 1,\;\lambda_{P3} = \frac{1}{{\lambda_{P1} }},$$

where *λ*_*P*1_*, λ*_*P*2_ and *λ*_*P*3_ represent the pre-stretch in the *x*, *y*, and *z* directions, respectively.

When a voltage load is applied, the deformation of the DE membrane in three main directions can be expressed as:

2 $$\lambda_{1} = \frac{L}{{L_{0} }},\;\lambda_{2} = 1,\;\lambda_{3} = \frac{1}{{\lambda_{1} }},$$

where *λ*_1_*, λ*_2_ and *λ*_3_ represent the actual stretch in the *x*, *y*, and *z* directions under the pre-stretch and voltage loads, respectively.

The length *L* of the DE membrane varies with the actual bending angle *θ* of the frame as follows:^53^

3 $$L = \frac{2C}{\theta }\sin \left( {\frac{\theta }{2}} \right).$$

Substitute Eq. (3) into Eq. (2), the actual stretch state of the DE membrane can be rewritten as:

4 $$\lambda_{1} = \frac{2C}{{\theta L_{0} }}\sin \left( {\frac{\theta }{2}} \right),\;\lambda_{2} = 1,\;\lambda_{3} = \frac{1}{{\lambda_{1} }}.$$

**Figure 3:**
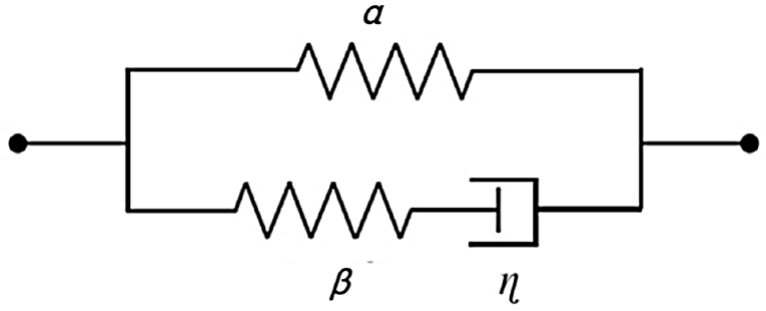
Zener rheological model.

DE usually exhibits significant viscoelasticity. In this paper, the Zener rheological model is used to describe the viscoelasticity behavior of DE, as shown in Fig. 3. The rheological model consists of two parallel elements: one element consists of an ideal spring; while the other element consists of an ideal spring and a damper (i.e. a Maxwell element). The in-plane deformation in *x* and *y* directions of the DE membrane is represented by *λ*_1_ and *λ*_2_, respectively. When the deformation reaches equilibrium state, the deformation amount in the two elements of the rheological model is equal. For spring *α*, its deformation in *x* and *y* directions can be expressed as *λ*_1_ and *λ*_2_. Similarly, for dampers, the stretch ratio in *x* and *y* directions can be expressed as *ξ*_1_ and *ξ*_2_. According to the multiplicative law, the state of deformation of spring *β* can be described by stretches *λ*_1_*ξ*_1_^−1^, *λ*_2_*ξ*_2_^−1^ and *λ*_1_^−1^*λ*_2_^−1^*ξ*_1_*ξ*_2_ in *x*, *y* and *z* directions, respectively. The deformation process of the Zener model is as follows: when there is no external force at both ends of the model, the system remains in equilibrium; when the left end of the system is given a fixed constraint, and the right end is subjected to displacement, the springs *α* and *β* will undergo transient deformation and the damping will not be able to undergo deformation in a relatively short period of time, at which time all the initial deformations of the model will be provided by the springs; and as time passes, the damping in the system will begin to deform slowly, and the springs will undergo a restitution to reach a certain equilibrium state at last to complete the stress relaxation process.

When analyzing the dynamic properties of DE membranes, the deformation of its active part near the equilibrium position is not significant, and the strain stiffening phenomenon under large deformation does not need to be considered. Therefore, the neo-Hookean model is chosen to describe the strain energy. Thus, the strain energy density function can be expressed as:

5 $$W = \frac{{\mu_{\alpha } }}{2}(\lambda_{1}^{2} + \lambda_{2}^{2} + \lambda_{1}^{ - 2} \lambda_{2}^{ - 2} - 3) + \frac{{\mu_{\beta } }}{2}(\lambda_{1}^{2} \xi_{1}^{ - 2} + \lambda_{2}^{2} \xi_{2}^{ - 2} + \lambda_{1}^{ - 2} \lambda_{2}^{ - 2} \xi_{1}^{2} \xi_{2}^{2} - 3),$$

where *μ*_*α*_ and *μ*_*β*_ are the shear moduli of spring *α* and spring *β* in the rheological model, respectively.

The special boundary conditions of the pure shear model satisfy *λ*_2_ = *ξ*_2_ = *λ*_*P*2_ = 1, then there is the following relationship:

6 $$\lambda_{2}^{2} \xi_{2}^{ - 2} { = }\lambda_{2}^{ - 2} \xi_{2}^{2} { = }1.$$

Let *λ*_1_ = *λ*, *ξ*_1_ = *ξ* to simplify Eq. (5), the strain energy density function can be given as:

7 $$W = \frac{{\mu_{\alpha } }}{2}(\lambda^{2} + \lambda^{ - 2} - 2) + \frac{{\mu_{\beta } }}{2}(\lambda^{2} \xi^{ - 2} + \lambda^{ - 2} \xi^{2} - 2).$$

The next section will investigate the energy generated by DE membranes during electrical treatment.

### DE membrane electrostatic energy

In order to consider the effect of electrostatic pressure generated by an external electric field on the strain energy of DE, this paper adopts an ideal dielectric model to consider the electrostatic energy, which is represented as:

8 $$W_{ela} = - \frac{1}{2}\varepsilon E^{2} \lambda_{1}^{2} \lambda_{2}^{2} = - \frac{1}{2}\varepsilon_{0} \varepsilon_{{\text{r}}} E^{2} \lambda^{2} ,$$

where *ε* = *ε*_0_*ε*_*r*_ represents the dielectric constant of DE, *ε*_0_ represents the dielectric constant, *ε*_*r*_ represents the influence of temperature on the dielectric constant. *E* represents the applied electric field strength on the DE membrane. The negative sign represents energy supplied from the outside to the DE membrane.

In addition to considering the pre-stretch factor, the influence of external environmental temperature on the performance of DEMES is also considered. Therefore, the influence of temperature on the dielectric constant of DE membranes should be considered here. The dielectric constant of DE membranes can be expressed as a function of temperature and deformation:^54^

9 $$\varepsilon_{r} \left( {\lambda ,\;T} \right) = \left( {\varepsilon_{\infty } + \frac{A}{T}} \right)\left( {1 + a(\lambda - 1) + b(\lambda - 1)^{2} + c(\lambda - 1)^{3} } \right),$$

where *T* is the temperature, *ε*_*∞*_, *A*, *a*, *b* and *c* are constants. The values of the constants are given as *ε*_*∞*_ = 2.1, *A* = 960, *a* =  − 0.1658, *b* = 0.04086 and *c* =  − 0.003027^54^. The next section will investigate the thermal contribution of DE membranes during operation.

#### Thermal contribution energy of DEMES

Considering the temperature effect factor, the free energy density function of the DE membrane should include a portion of the energy contributed by the heat:

10 $$W_{h} = \rho c_{0} \left[ {T - T_{0} - T\ln \left( {\frac{T}{{T_{0} }}} \right)} \right],$$

where *c*_0_ is the specific heat capacity of DE, *ρ* is the density of DE, *T*_0_ is the reference temperature. The next section will study the free energy strain equation of DE membranes.

### Free energy density function of DE membrane

The free energy density function of DE membrane can be represented by a combination of strain energy, electrostatic energy, and thermal contribution energy:

11 $$\begin{aligned} W_{DE} & = \frac{{\mu_{\alpha } }}{2}\left( {\lambda^{2} + \lambda^{ - 2} - 2} \right) + \frac{{\mu_{\beta } }}{2}\left( {\lambda^{2} \xi^{ - 2} + \lambda^{ - 2} \xi^{2} - 2} \right) \\ & \quad - \frac{1}{2}\varepsilon_{0} \varepsilon_{{\text{r}}} \left( {\lambda ,T} \right)E^{2} \lambda^{2} + \rho c_{0} \left[ {T - T_{0} - T\ln \left( {\frac{T}{{T_{0} }}} \right)} \right]. \\ \end{aligned}$$

The next section will establish the dynamic governing equation of DEMES.

## Dynamic governing equations

In order to characterize the nonlinear dynamics of DEMES under the action of pre-stretch and temperature effects, the non-conservative system Eulerian–Lagrange modelling approach is used, which follows the principle of least action, and can be expressed as follows:

12 $$\frac{{\text{d}}}{{{\text{dt}}}}\left( {\frac{\partial L}{{\partial \dot{\theta }}}} \right) - \frac{\partial L}{{\partial \theta }} + \frac{\partial D}{{\partial \dot{\theta }}}{ = }0,$$

where *D* represents the dissipation function, $\dot{\theta }$ represents the derivative of the bending angle *θ* of DEMES relative to time, and *L* represents the Lagrange function of the system. The Lagrangian of the DEMES actuator system, which represents the difference between the total kinetic energy and the total potential energy of DEMES, can be given as:

13 $$L{ = }T - U,$$

where *T* represents the total kinetic energy and *U* represents the total potential energy of DEMES.

### Total potential energy of DEMES

The total potential energy of DEMES consists of two parts: the potential energy of the flexible frame *U*_*F*_ and the potential energy of the DE membrane *U*_*DE*_. The flexible frame can be regarded as a bending spring with a constant bending moment, and the potential energy is given as follows:

14 $$U_{F} { = }\frac{1}{2}K_{{\text{b}}} \theta^{2} = \frac{{Y_{f} bd^{3} }}{24C}\theta^{2} ,$$

where *K*_b_ is the bending stiffness of the compliant frame and *Y*_*f*_ is the Young’s modulus of the flexible frame.

According to the assumption of incompressibility, the potential energy of DE membrane can be expressed as:

15 $$U_{DE} = V_{DE} \times W_{DE} ,$$

where *V*_*DE*_ is the volume of the DE membrane.

Due to the fact that DEMES consists of two parts: DE membrane and flexible frame, and it is known that Eqs. (14), (15) represent the potential energies of flexible frame and DE membrane. The total potential energy of DEMES is the sum of the potential energies of DE membrane and flexible frame, which can be expressed as:

16 $$\begin{aligned} U & = U_{F} + U_{DE} = \frac{{K_{b} }}{2}\theta^{2} \\ & \quad + L_{0} W_{0} t_{0} \left[ \begin{gathered} \frac{{\mu_{\alpha } }}{2}\left( {\lambda^{2} + \lambda^{ - 2} - 2} \right) + \frac{{\mu_{\beta } }}{2}\left( {\lambda^{2} \xi^{ - 2} + \lambda^{ - 2} \xi^{2} - 2} \right) \hfill \\ - \frac{1}{2}\varepsilon_{0} \varepsilon_{{\text{r}}} \left( {\lambda ,T} \right)E^{2} \lambda^{2} + \rho c_{0} \left[ {T - T_{0} - T\ln \left( {\frac{T}{{T_{0} }}} \right)} \right] \hfill \\ \end{gathered} \right]. \\ \end{aligned}$$

This article specifies ABS material as the flexible frame part of DEMES, and the elastic modulus of this material is related to temperature. Meng obtained the elastic modulus of ABS plastic at different temperatures through experiments^55^, and the approximate relationship between the elastic modulus of ABS material and ambient temperature can be expressed as follows (unit: MPa):

17 $$Y_{f} \left( T \right) = - 20T + 7258.552,\quad 240\;{\text{K}} \le T \le 360\;{\text{K}}.$$

### Total kinetic energy of DEMES

Since the weight of the DE membrane is much smaller than that of the flexible frame, the kinetic energy of the DE membrane can be neglected as well as reducing the flexible frame to a simple rotating hinge. Therefore, the total kinetic energy of the DEMES is expressed as follows:

18 $$T{ = }\frac{1}{2}I\left( {\frac{{{\text{d}}\theta }}{{{\text{d}}t}}} \right)^{2} ,$$

where *I* is the rotational inertia, *θ* is the bending angle of DEMES.

### Rayleigh dissipation function

The Rayleigh dissipation function is used to characterize the viscous damping, which can be expressed as:

19 $$D\left( {\dot{\xi }} \right){ = }\frac{1}{2}\eta \dot{\xi }^{2} V,$$

where *η* is the viscosity parameter, $\dot{\xi }$ is the time derivative of viscous stretch in the pre-stretched principal direction.

Let* μ*_*α*_ = *μ* and *μ*_*β*_ = *βμ*, substituting Eqs. (16)–(19) into Eq. (12), the nonlinear dynamic governing equation of DEMES considering the pre-stretch and temperature factors can be written as:

20 $$\begin{gathered} \frac{I}{{L_{0} W_{0} t_{0} \mu }}\frac{{{\text{d}}^{2} \theta }}{{{\text{d}}t^{2} }} + \frac{{K_{b} \theta }}{{L_{0} W_{0} t_{0} \mu }} + 2\left( {\lambda_{P1} } \right)^{2} \sin \frac{\theta }{2}\left[ {\frac{{\theta \cos \frac{\theta }{2} - 2\sin \frac{\theta }{2}}}{{\theta^{3} }}} \right] \hfill \\ \quad + \frac{1}{8}\left( {\frac{1}{{\lambda_{P1} }}} \right)^{2} \left[ {\frac{{2\theta \sin \frac{\theta }{2} - \theta^{2} \cos \frac{\theta }{2}}}{{\sin^{3} \frac{\theta }{2}}}} \right] + 2\beta \left( {\lambda_{P1} } \right)^{2} \sin \frac{\theta }{2}\left[ {\frac{{\theta \cos \frac{\theta }{2} - 2\sin \frac{\theta }{2}}}{{\theta^{3} }}} \right]\xi^{ - 2} \hfill \\ \quad + \frac{\beta }{8}\left( {\frac{1}{{\lambda_{P1} }}} \right)^{2} \left[ {\frac{{2\theta \sin \frac{\theta }{2} - \theta^{2} \cos \frac{\theta }{2}}}{{\sin^{3} \frac{\theta }{2}}}} \right]\xi^{2} - 2\left( {\lambda_{P1} } \right)^{2} \sin \frac{\theta }{2}\left[ {\frac{{\theta \cos \frac{\theta }{2} - 2\sin \frac{\theta }{2}}}{{\theta^{3} }}} \right]\frac{{\varepsilon_{0} \varepsilon_{r} E^{2} }}{\mu } \hfill \\ \quad + \frac{4}{\mu }\left( {\frac{1}{{\lambda_{P1} }}} \right)^{3} \sin^{2} \frac{\theta }{2}\left[ {\frac{{\theta \cos \frac{\theta }{2} - 2\sin \frac{\theta }{2}}}{{\theta^{4} }}} \right]\varepsilon_{0} \left( {\varepsilon_{\infty } + \frac{A}{T}} \right)E^{2} \left( {a + 2b + 3c} \right) = 0. \hfill \\ \end{gathered}$$

Rewrite Eq. (20) in dimensionless form, which can be given as:

21 $$\begin{gathered} \frac{{{\text{d}}^{2} \theta }}{{{\text{d}}\tau^{2} }} + K_{bb} \theta + 2\left( {\lambda_{P1} } \right)^{2} \sin \frac{\theta }{2}\left[ {\frac{{\theta \cos \frac{\theta }{2} - 2\sin \frac{\theta }{2}}}{{\theta^{3} }}} \right] + \frac{1}{8}\left( {\frac{1}{{\lambda_{P1} }}} \right)^{2} \left[ {\frac{{2\theta \sin \frac{\theta }{2} - \theta^{2} \cos \frac{\theta }{2}}}{{\sin^{3} \frac{\theta }{2}}}} \right] \hfill \\ \quad + 2\beta \left( {\lambda_{P1} } \right)^{2} \sin \frac{\theta }{2}\left[ {\frac{{\theta \cos \frac{\theta }{2} - 2\sin \frac{\theta }{2}}}{{\theta^{3} }}} \right]\xi^{ - 2} + \frac{\beta }{8}\left( {\frac{1}{{\lambda_{P1} }}} \right)^{2} \left[ {\frac{{2\theta \sin \frac{\theta }{2} - \theta^{2} \cos \frac{\theta }{2}}}{{\sin^{3} \frac{\theta }{2}}}} \right]\xi^{2} \hfill \\ \quad - 2\left( {\lambda_{P1} } \right)^{2} \sin \frac{\theta }{2}\left[ {\frac{{\theta \cos \frac{\theta }{2} - 2\sin \frac{\theta }{2}}}{{\theta^{3} }}} \right]e_{1}^{2} + 4\left( {\lambda_{P1} } \right)^{3} \sin^{2} \frac{\theta }{2}\left[ {\frac{{\theta \cos \frac{\theta }{2} - 2\sin \frac{\theta }{2}}}{{\theta^{4} }}} \right]e_{2}^{2} = 0, \hfill \\ \end{gathered}$$

where $\tau = t\sqrt {{{\mu L_{0} W_{0} t_{0} } \mathord{\left/ {\vphantom {{\mu L_{0} W_{0} t_{0} } I}} \right. \kern-0pt} I}}$ is the dimensionless time, $K_{bb} = {{K_{b} } \mathord{\left/ {\vphantom {{K_{b} } {\left( {L_{0} W_{0} t_{0} \mu } \right)}}} \right. \kern-0pt} {\left( {L_{0} W_{0} t_{0} \mu } \right)}}$ is the dimensionless stiffness, $e_{1} = E\sqrt {{{\varepsilon_{0} \varepsilon_{r} } \mathord{\left/ {\vphantom {{\varepsilon_{0} \varepsilon_{r} } \mu }} \right. \kern-0pt} \mu }}$ and $e_{2} = E\sqrt {\frac{{\varepsilon_{0} }}{\mu }\left( {\varepsilon_{\infty } + \frac{A}{T}} \right)\left( {a + 2b + 3c} \right)}$ are the dimensionless electric fields.

By combining the Lagrange function *L* and dissipation function *D* of the system, we can express the following differential evolution equation as:

22 $$\frac{{\text{d}}}{{{\text{dt}}}}\left( {\frac{\partial L}{{\partial \dot{\xi }}}} \right) - \frac{\partial L}{{\partial \xi }} + \frac{\partial D}{{\partial \dot{\xi }}}{ = }0,$$

Substituting Eqs. (16)–(19) in Eq. (22), the final differential evolution equation can be expressed as:

23 $$\frac{{{\text{d}}\xi }}{{{\text{d}}\tau }}\sqrt {\frac{{\mu L_{0} W_{0} t_{0} }}{I}} + \frac{\mu \beta }{\eta }\left[ {\frac{{L_{0}^{2} \theta^{2} \xi }}{{4C^{2} \sin^{2} \left( {\frac{\theta }{2}} \right)}} - \frac{{4C^{2} \sin^{2} \left( {\frac{\theta }{2}} \right)\xi^{ - 3} }}{{L_{0}^{2} \theta^{2} }}} \right]{ = }0.$$

The viscosity and elastic modulus of DE are both related to ambient temperature and can be expressed as *η*(*T*) and *μ*(*T*). Then, the relaxation time *S*(*T*) of DE can be expressed as:

24 $$s(T) = \frac{\eta (T)}{{\mu (T)}} = 63.64 + 1.766 \times 10^{ - 5} \exp \left( \frac{3850}{T} \right).$$

The final differential evolution equation of DEMES can be expressed as:

25 $$\frac{{{\text{d}}\xi }}{{{\text{d}}\tau }}\sqrt {\frac{{\mu L_{0} W_{0} t_{0} }}{I}} + \frac{\beta }{s(T)}\left[ {\frac{{L_{0}^{2} \theta^{2} \xi }}{{4C^{2} \sin^{2} \left( {\frac{\theta }{2}} \right)}} - \frac{{4C^{2} \sin^{2} \left( {\frac{\theta }{2}} \right)\xi^{ - 3} }}{{L_{0}^{2} \theta^{2} }}} \right]{ = }0.$$

When conducting the numerical calculation part of this study, reasonable initial conditions should be set. When the pre-stretched DE membrane is initially connected to the flexible frame, the flexible frame should be considered horizontal, that is, at *τ* = 0, the bending angle of the DEMES is 0, the angular velocity of motion is 0, and the non-elastic deformation is the initial pre-stretch ratio. Finally, the initial conditions for DEMES are as follows:

26 $$\theta_{\tau = 0} = 0,\;\frac{d\theta }{{d\tau }}_{\tau = 0} = 0,\;\xi_{\tau = 0} = \lambda_{P1} .$$

The next section will use the established DEMES dynamic governing equation for numerical simulation analysis of dynamic characteristics.

## Numerical simulation

In this paper, the operating system used is Microsoft Windows 11 and the simulation platform chosen is MATLAB R2020b. The theoretical model of DEMES obtained in the above section is computed using the ODE differential equation solver. The effect of pre-stretch on the dynamic performance of DEMES is discussed through numerical simulations. To examine the impact of pre-stretch on the mechanical behavior of DEMES, different pre-stretch values were considered. Other important material parameters such as shear modulus ratio constant of the spring *β* = 2.5.

The next section will investigate the dynamic characteristics of DEMES under the influence of individual pre-stretch. It should be noted that for the anisotropy of DE membranes^56^, it can also significantly control the driving behavior. As this article mainly studies the influence of pre-stretch factors on the dynamic characteristics of DEMES, the following research focuses on the isotropy of DE membranes.

### Analysis of the effect of pre-stretch on DEMES under voltage-free driving conditions

This section focuses on analyzing the response law of DEMES in the process of reaching the final equilibrium state when different pre-stretch loads are applied without driving voltage. In order to avoid the influence of ambient temperature on the dynamic performance of the DEMES, the elastic modulus and dielectric constant of the system are set to the values at 26 °C (299 K). The electric field strength is set to *E* = 0. In order to investigate the effect of pre-stretch on the equilibrium state of DEMES, different values of pre-stretch ratio are set as *λ*_*P*1_ = 2.0, 2.5, 3.0, 3.5, 4.0, 4.5 and 5.0, respectively.

**Figure 4:**
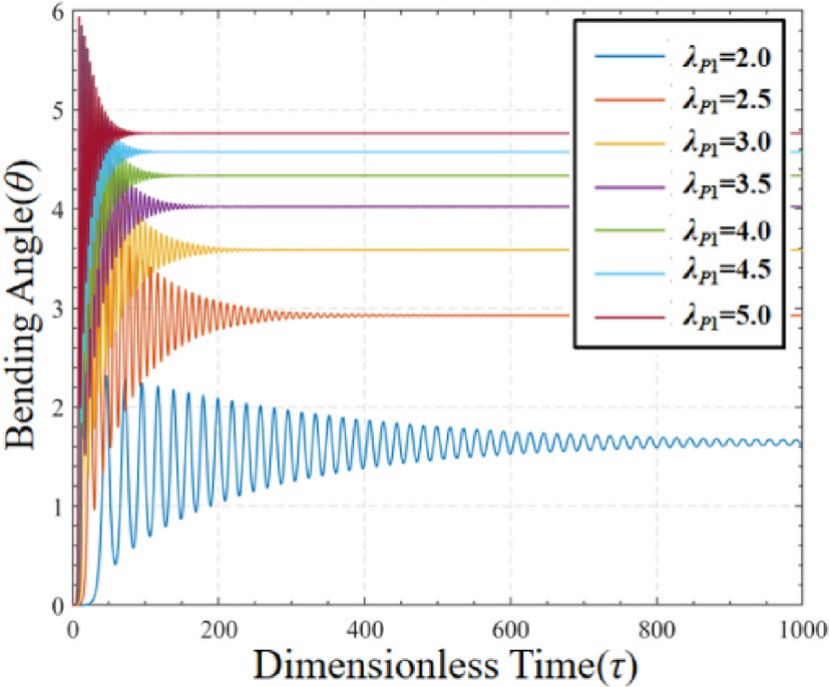
Bending angle of DEMES with different pre-stretch ratios under voltage-free driving conditions.

The simulation results for the response of pre-stretch under voltage-free driving conditions are presented in Fig. 4. The figure illustrates that when a pre-stretch load is applied to the DEMES without driving voltage, the system oscillates before attaining equilibrium state, with the magnitude of oscillation diminishing over time. The initial horizontal state leads to the partial release of elastic potential energy when the pre-stretched DE membrane is affixed to the horizontal flexible frame, resulting in oscillation. In addition, it can be observed that the final equilibrium angle of the DEMES increases with the increase of the pre-stretch *λ*_*P*1_. When the pre-stretch *λ*_*P*1_ increases, the pre-stretch stress inside the DE membrane becomes progressively larger, and therefore leads to an increase in the bending angle of the DEMES. It can also be seen from the results that the time required for the system to reach the equilibrium state is also less when the pre-stretch *λ*_*P*1_ is higher. This is due to the acceleration of the deformation process caused by the larger pre-stretch stresses as *λ*_*P*1_ increases. The above research results indicate that the influence of pre-stretch on the equilibrium deformation of DEMES cannot be ignored.

**Figure 5:**
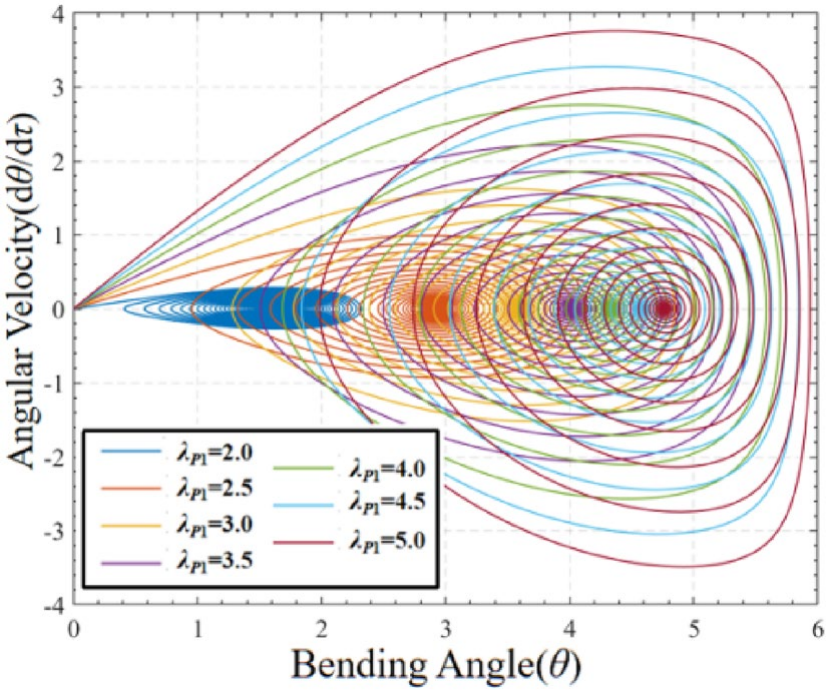
Phase paths of DEMES with different pre-stretch ratios under voltage-free driving conditions.

In order to further investigate the effect of pre-stretch on the dynamic characteristics of DEMES, the phase paths of pre-stretch response angle versus angular velocity of DEMES are investigated for different stretch ratios of *λ*_*P*1_ = 2.0, 2.5, 3.0, 3.5, 4.0, 4.5 and 5.0, as shown in Fig. 5. It is obvious from the figure that DEMES oscillates around the equilibrium position and finally reaches the equilibrium state at different pre-stretch ratios. Specifically, the DEMES under conditions of higher pre-stretch ratios experience more violent oscillations in order to reach the equilibrium position. With increasing pre-stretch ratio, the final equilibrium angle of DEMES enlarges, accompanied by a notable rise in angular velocity throughout the oscillation process towards equilibrium. Consequently, we can conclude that an increase in the pre-stretch ratio intensifies the magnitude of DEMES oscillation in response to pre-stretch. It can be concluded that the increase in the pre-stretch ratio exacerbates the intensity of the DEMES oscillations. The reason for this phenomenon is that the rise in the pre-stretch ratio increases the pre-stretch stresses inside the DE membrane and the flexible frame.

**Figure 6:**
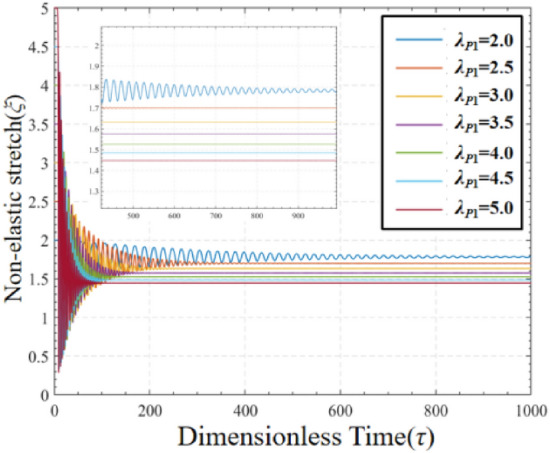
The non-elastic deformation of DEMES with different pre-stretch ratios under voltage-free driving conditions.

In order to study the effect of different pre-stretch ratios on the non-elastic deformation *ξ* of DEMES, a graph of the variation of non-elastic deformation *ξ* over time under different pre-stretch ratios is plotted in Fig. 6. When the pre-stretch ratio is 2.0, the value of non-elastic deformation *ξ* oscillates over time and eventually stabilizes. As the pre-stretch ratio increases, the final stable value of non-elastic deformation *ξ*decreases. At higher pre-stretch ratios, such as 3.0–5.0 pre-stretch ratios, the amplitude of non-elastic deformation *ξ* changes is significantly reduced compared to the changes at lower pre-stretch ratios. For all pre-stretch ratios, the time required to reach the final stable value of non-elastic deformation *ξ* is roughly equal. When DEMES is in the equilibrium state, the non-elastic deformation *ξ* is equal to the elastic stretch *λ*_*p*_. Hence, the non-elastic deformation *ξ* is obtained to be 1.78, 1.7, 1.63, 1.57, 1.53, 1.48 and 1.45 for pre-stretch *λ*_*P*1_ = 2.0, 2.5, 3.0, 3.5, 4.0, 4.5 and 5.0, respectively. This result is in agreement with the values of non-elastic deformation *ξ* in the steady state in Fig. 6. These results can be used as initial values for the following dynamic analysis.

### Analysis of the effect of pre-stretch on DEMES under voltage driving conditions

#### Analysis of the effect of pre-stretch on DEMES under DC driving conditions

**Figure 7:**
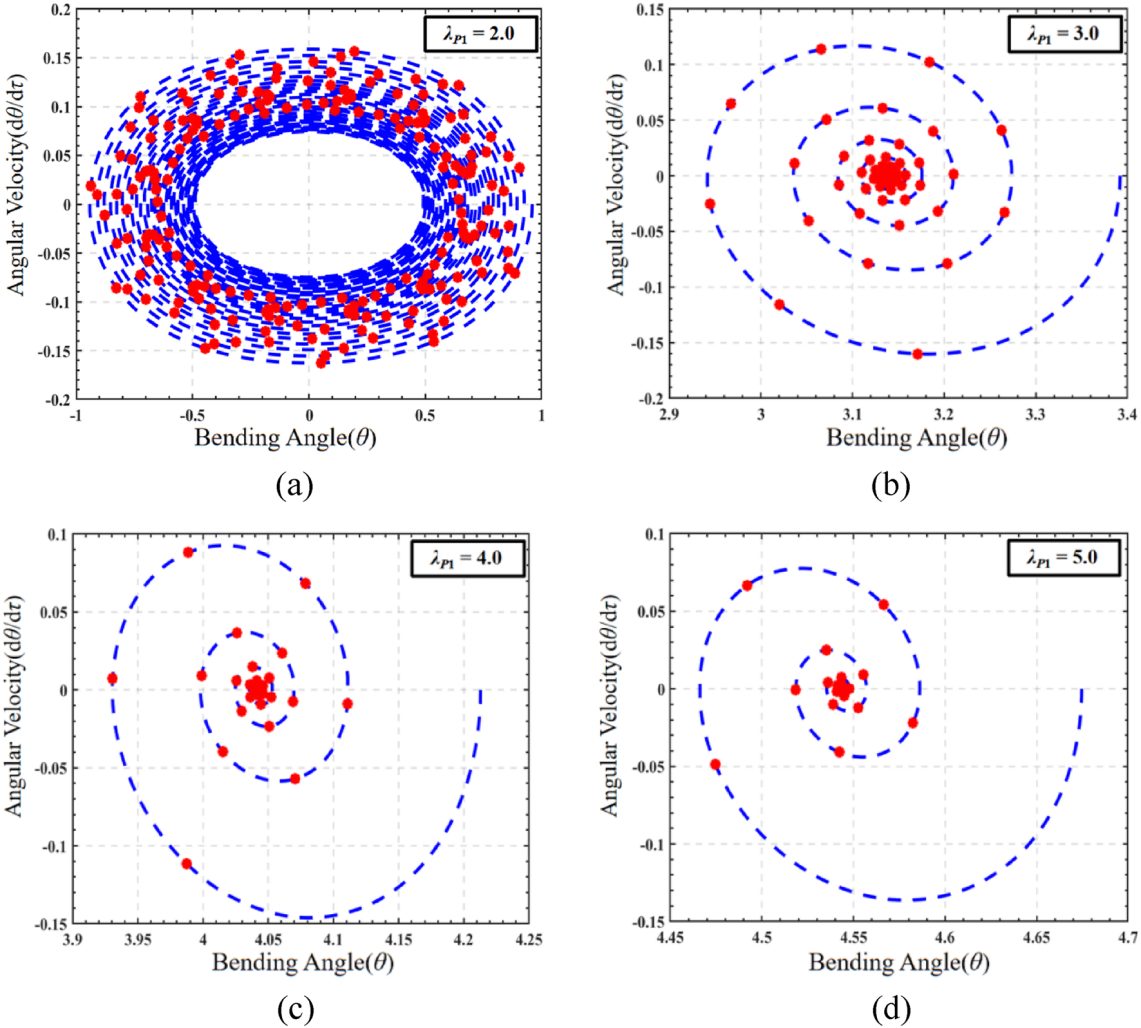
The phase paths and Poincaré maps of DEMES with different pre-stretch ratios under DC driving conditions. (**a**) *λ*_*P*1_ = 2.0, (**b**) *λ*_*P*1_ = 3.0, (**c**)* λ*_*P*1_ = 4.0 and (**d**) *λ*_*P*1_ = 5.0.

When analyzing the influence of pre-stretch load on the dynamic characteristics of DEMES under DC driving conditions, the initial conditions should be set as the final equilibrium state response results in Sect. “Analysis of the effect of pre-stretch on DEMES under voltage driving conditions”. Given a DC voltage of *V*_*dc*_ = 5000 V. In order to evaluate the impact of different pre-stretch ratios on the dynamic performance of DEMES under DC field, the phase paths and corresponding Poincaré maps of the dynamic response of DEMES under pre-stretch *λ*_*P*1_ = 2.0, 3.0, 4.0, and 5.0 are analyzed, as shown in Fig. 7. When the pre-stretch ratio is 2.0, the response phase path of DEMES shows uniform oscillation and does not significantly decay over time, as shown in Fig. 7a. Correspondingly, the Poincaré map at a pre-stretch ratio of 2.0 is a closed loop curve, reflecting that the oscillation of DEMES is quasi periodic, as shown in Fig. 7a. At a pre-stretch ratio of 3.0, DEMES experienced a maximum decrease in angular velocity, leading to a more pronounced attenuation trend. The angular velocity decreases faster during the oscillation process. The obvious divergent state in the Poincaré map indicates the occurrence of non-periodic oscillations in DEMES, as shown in Fig. 7b. For pre-stretch ratios of 4.0 and 5.0, the overall oscillation trend is similar to before, the oscillation of DEMES continues to decay until it completely disappears, and the angular velocity decreases to 0, as shown in Fig. 7c and d. In summary, the pre-stretch ratio has a significant impact on the reconstruction of DEMES from an equilibrium state to a new equilibrium state under DC excitation. When the pre-stretch ratio is low, the pre-stretch stress in the DE membrane is relatively low, and therefore the oscillations persist for a longer time when excited by a voltage. As the pre-stretch ratio increases, the pre-stretch stress in the DE membrane is enhanced, leading to a weakening of the oscillation amplitude during the deformation process. In the end, this oscillation decays to 0 with deformation, and the Poincaré map takes on the form of a centroid. Compared with before the voltage was applied, the oscillation process of DEMES at various pre-stretch ratios showed two states: periodic oscillation and non-periodic oscillation. This is noteworthy because non-periodic oscillation is difficult to control and is detrimental to the stability of the operation.

**Figure 8:**
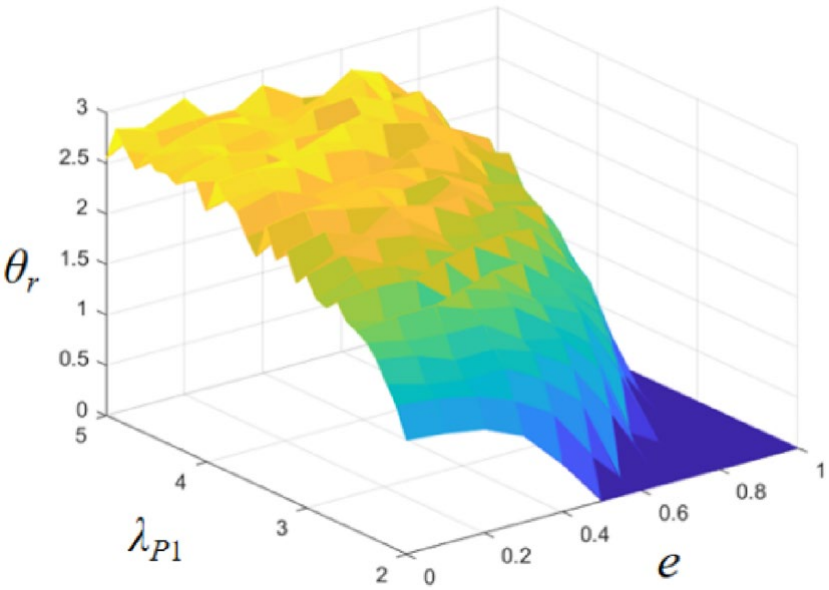
Bending angle fluctuation range of DEMES for different values of pre-stretch ratio and applied DC voltage.

In order to investigate the effect of pre-stretch ratio and DC voltage on the bending angle of the DEMES, the fluctuation range of the DEMES is plotted versus the variation of the pre-stretch ratio and DC voltage, as shown in Fig. 8. In the figure, *e* represents the dimensionless electric field, and *θ*_*r*_ represents the fluctuation range defined as the difference between the maximum oscillation angle and the minimum oscillation angle. During the increase of the pre-stretch ratio *λ*_*P*1_ from 2.0 to 4.0, the fluctuation of DEMES is correspondingly increased. This is because an increase in the pre-stretch ratio leads to an increase in the pre-stretch stress in the DE membrane, which increases the initial bending equilibrium angle and the elastic potential energy of DEMES, leading to a large fluctuation in its yield bending angle when loaded with a DC voltage load. Therefore, when subjected to DC voltage loading, larger fluctuations in the bending angle are observed. At the same time, an increase in voltage leads to an enhancement of the stretching ability of the DE membrane, and the frame tends to flatten when the voltage reaches a high level and the pre-stretch ratio is at a low level. The trend of the 3D rendering intuitively reflects the influence of voltage and pre-stretch ratio on DEMES deformation.

### Analysis of the effect of pre-stretch on DEMES under AC driving conditions

When analyzing the effect of pre-stretch ratios on the dynamic characteristics of DEMES under AC driving conditions, the initial conditions should be set to the equilibrium state results of Sect. “Analysis of the effect of pre-stretch on DEMES under voltage driving conditions”. The applied AC voltage can be given as:

27 $$V = V_{dc} + V_{ac} \sin \left( {\hat{\omega }\tau } \right){.}$$

where *V*_*dc*_ is the DC voltage, *V*_*ac*_ is the amplitude of AC voltage and $\hat{\omega } = \omega \sqrt {{I \mathord{\left/ {\vphantom {I {\left( {\mu L_{0} W_{0} t_{0} } \right)}}} \right. \kern-0pt} {\left( {\mu L_{0} W_{0} t_{0} } \right)}}}$ is the dimensionless frequency of the AC voltage, where *ω* is the frequency of excitation. The DC voltage *V*_*dc*_ and the amplitude of AC voltage *V*_*ac*_ are set as 1000 V and 5000 V, respectively.

The following figure shows the amplitude frequency response curves of DEMES under different pre-stretch ratios. The dimensionless frequency *ω* range is set to 0–4, and the pre-stretch ratio *λ*_*P*1_ = 2.0, 3.0, 4.0, and 5.0.

The results show that the DEMES response presents an obvious law of change at different AC frequencies. Both the resonant frequency and peak value of DEMES decrease with the increase of pre-stretch ratio. This is because the increased pre-stretch ratio leads to an increase in the pre-stretch stress inside the DE membrane, which requires only a smaller excitation frequency to trigger the resonance phenomenon.

**Figure 9:**
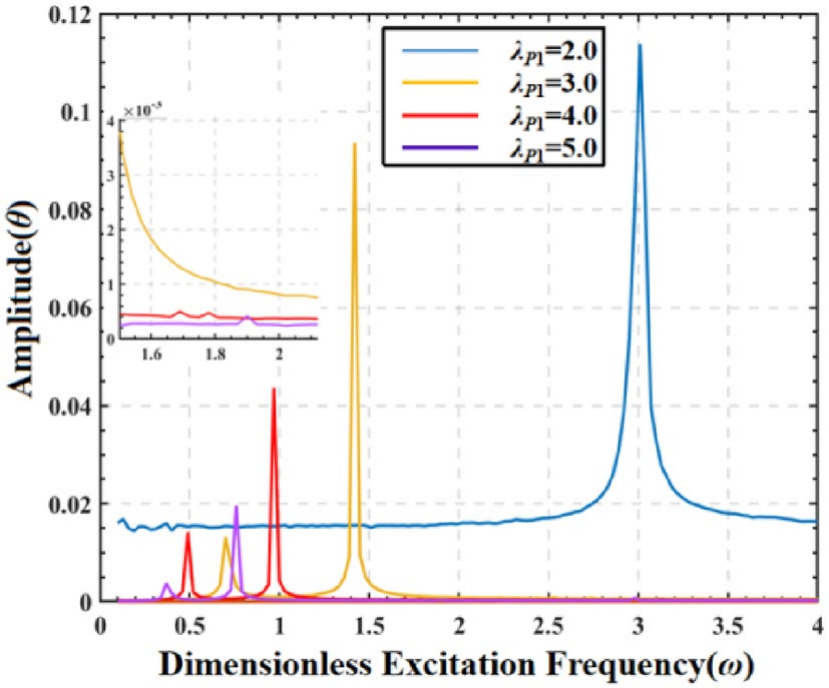
Amplitude frequency response of DEMES with different pre-stretch ratios under AC driving conditions.

**Figure 10:**
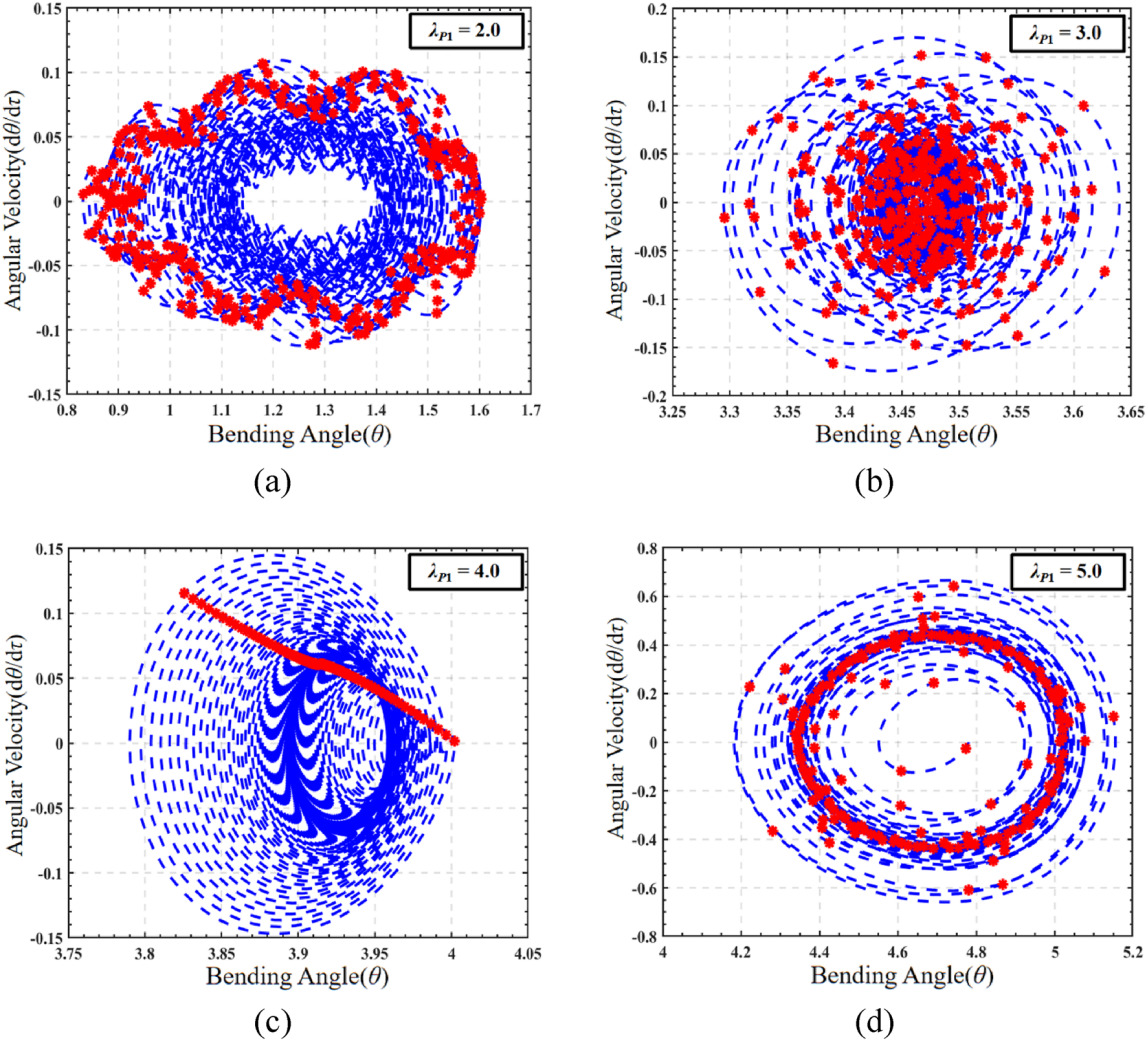
The phase paths and Poincaré maps of DEMES with different pre-stretch ratios under AC driving conditions. (**a**) *λ*_*P*1_ = 2.0, (**b**) *λ*_*P*1_ = 3.0, (**c**) *λ*_*P*1_ = 4.0 and (**d**) *λ*_*P*1_ = 5.0.

To investigate the impact of the pre-stretch on the dynamic response of DEMES, the phase map and Poincaré map of the system under AC electrical load are plotted, as illustrated in Fig. 10. Based on the AC sweep frequency information in Fig. 9, 1.0 is selected as the AC excitation frequency. At a pre-stretch ratio of 2.0, the phase path of DEMES exhibits a uniform oscillation form near the equilibrium angle, while the corresponding Poincaré map is in the form of a closed-loop curve, indicating that DEMES is undergoing quasi periodic motion, as shown in Fig. 10a. As the pre-stretch ratio increases to 3.0, the phase path of DEMES begins to exhibit a transition from uniform oscillation to non-uniform oscillation, while the Poincaré plot transitions from a stable closed-loop to a dispersion mode, indicating that DEMES is undergoing a transition from quasi periodic motion to non-periodic oscillation, as shown in Fig. 10b. When the pre-stretch ratio is 4.0, the phase path of DEMES tends to converge again to a horn shaped aggregation form. The Poincaré map shows a straight line, indicating the existence of periodic oscillation orbits in DEMES and a critical state of transition from non-periodic oscillation to periodic oscillation, as shown in Fig. 10c. When the pre-stretch ratio is 5.0, the phase path of DEMES exhibits a uniform oscillation form near the equilibrium angle, and the Poincaré plot shows a clear closed-loop state, indicating that DEMES is in a stable periodic oscillation state, as shown in Fig. 10d. The results show that compared with the phase path and Poincaré map of DEMES under DC load, the oscillation changes of DEMES under AC load are more diverse, experiencing a transition from periodic oscillation to non-periodic oscillation and then to periodic oscillation. This may be due to the varying degrees of resonance between the AC excitation signal and the DEMES when the structure is in different pre tension ratios, which promotes the oscillation of the structure.

The next section will investigate the dynamic characteristics of DEMES under the coupling effect of temperature and pre-stretch.

### Analysis of the coupling effect of pre-stretch and temperature on DEMES under voltage-free driving conditions

**Figure 11:**
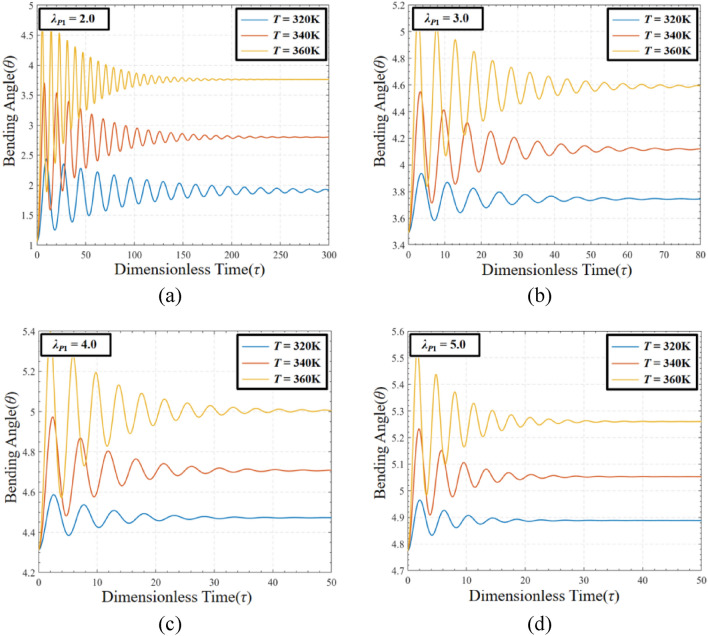
Bending angle of DEMES with different pre-stretch ratios and temperatures under voltage-free driving conditions. (**a**) *λ*_*P*1_ = 2.0, (**b**) *λ*_*P*1_ = 3.0, (**c**) *λ*_*P*1_ = 4.0 and (**d**) *λ*_*P*1_ = 5.0.

In order to study the effects of pre-stretch and temperature on the equilibrium state of DEMES, the temperature *T* = 320 K, *T* = 340 K and 360 K with different pre-stretch ratios (*λ*_*P*1_ = 2.0, 3.0, 4.0 and 5.0) are selected for analysis. The results are shown in Fig. 11. The figures show that the equilibrium angle increases with the improve of the temperature at the same pre-stretch ratio. This is mainly due to the decrease in stiffness of DEMES caused by the increase in temperature. For the same temperature, the equilibrium angle of DEMES increases with the rise of the pre-stretch ratio. This is the same as the results shown in Fig. 4. In addition, as the pre-stretch ratio increases, the oscillation time required for DEMES to reach equilibrium state decreases. This is mainly due to the increase in pre-stretch ratio leading to an increase in DE membrane pre-stretch stress, which plays a dominant role.

**Figure 12:**
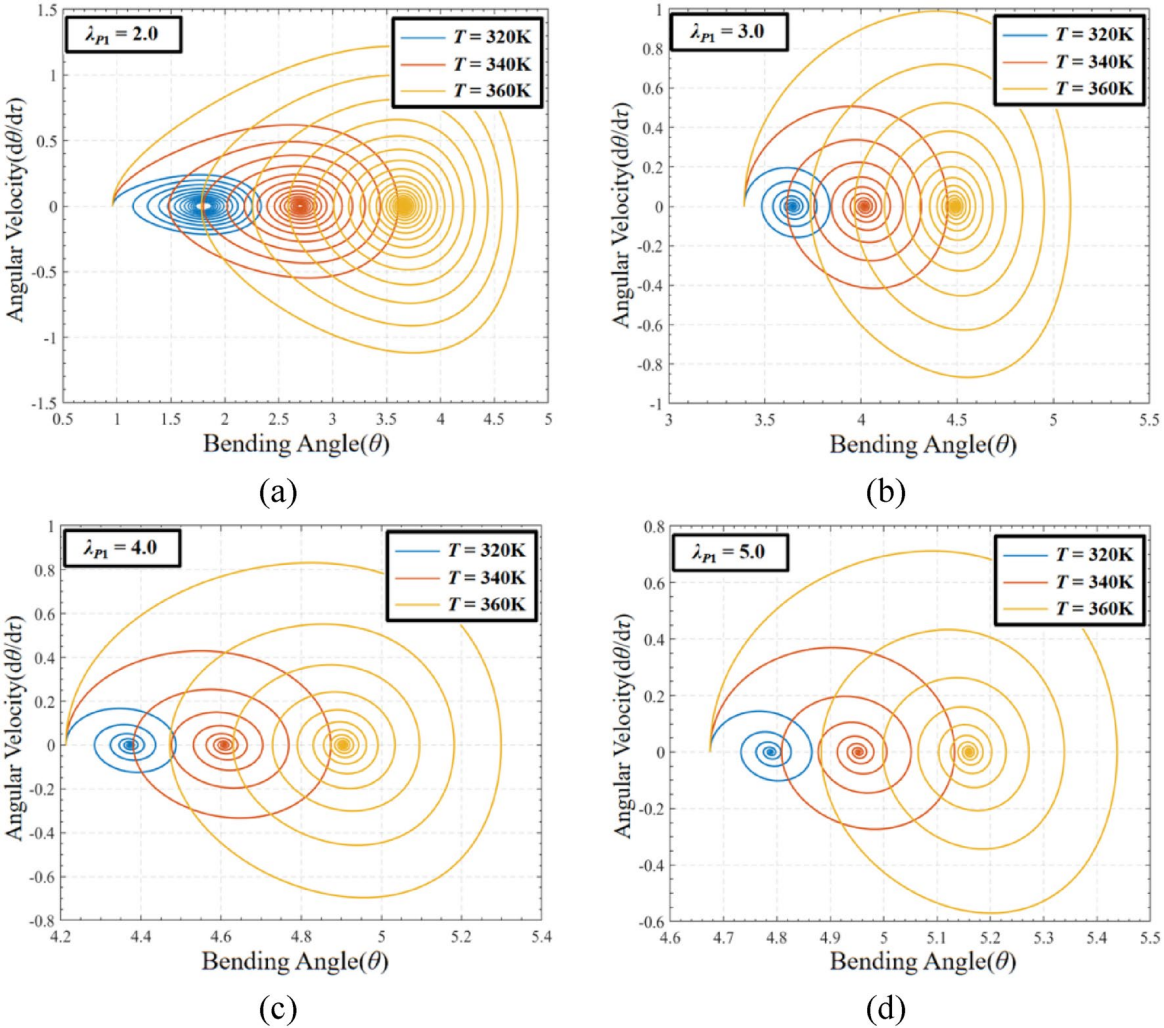
Phase paths of DEMES with different pre-stretch ratios and temperatures under voltage-free driving conditions. (**a**) *λ*_*P*1_ = 2.0, (**b**) *λ*_*P*1_ = 3.0, (**c**) *λ*_*P*1_ = 4.0 and (**d**) *λ*_*P*1_ = 5.0.

In order to evaluate the coupling effect of pre-stretch and temperature on DEMES under voltage-free driving conditions, the phase paths and the corresponding Poincaré maps of the dynamic response of DEMES with different pre-stretch ratios at temperature *T* = 320 K, 340 K and 360 K are analyzed, as shown in Fig. 12. In this case, the pre-stretch ratio *λ*_*P*1_ = 2.0, 3.0, 4.0 and 5.0.

Figure 12 shows the oscillation amplitude of DEMES under the coupling influence of pre-stretch load and temperature load. The results that when the pre-stretch ratio is 2.0 and the temperature is 320 K, the DEMES oscillates near the equilibrium angle without attenuation, ultimately reaching an equilibrium state. When the temperature at 340 K and 360 K, the DEMES experienced stronger oscillations, as shown in Fig. 12a. Figure 12b, c and d have the similar trends. As the pre-stretch ratio increases, the final equilibrium angle of DEMES significantly increases, and the maximum angular velocity during the oscillation process decreases. At the same time, the oscillation angular velocity finally stabilizes at 0, as shown in Fig. 12b, c, and d. It can be concluded that higher temperatures enhance the oscillation response of the DEMES, which is caused by the enhanced activity of the DE membrane and internal molecular chains within the flexible framework. In addition, the pre-stretch also affects the oscillation process of DEMES. When the pre-stretch ratio *λ*_*P*1_ = 2.0 and temperature *T* = 320, the equilibrium of DEMES is in a state of constant oscillation, with rigidity playing a dominant role. When the pre-stretch ratio *λ*_*P*1_ ≥ 3, DEMES exhibits similar oscillation modes at temperatures *T* = 320 K, 340 K and 360 K, and the oscillation angular velocity ultimately drops to zero, with viscoelasticity being the main factor.

**Figure 13:**
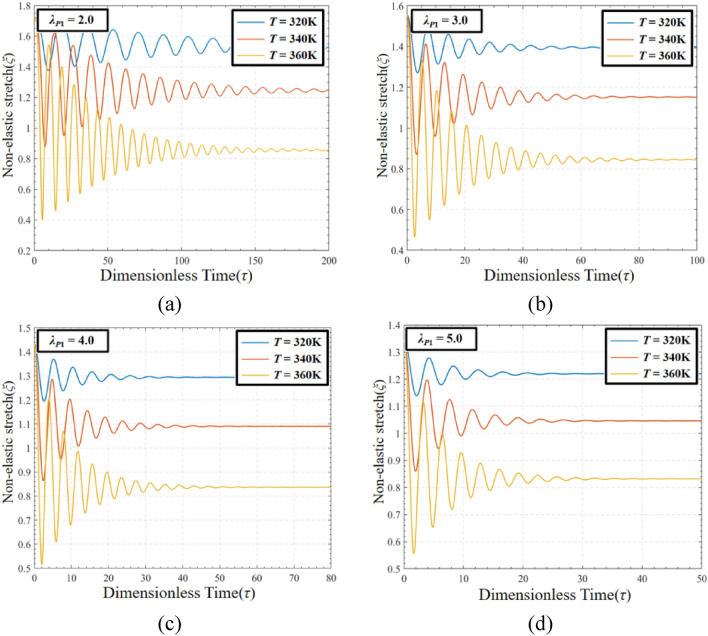
The non-elastic deformation of DEMES with different pre-stretch ratios and temperatures under voltage-free driving conditions. (**a**) *λ*_*P*1_ = 2.0, (**b**) *λ*_*P*1_ = 3.0, (**c**) *λ*_*P*1_ = 4.0 and (**d**) *λ*_*P*1_ = 5.0.

In order to evaluate the coupling effect of pre-stretch and temperature on DEMES under voltage-free driving conditions, the non-elastic deformation *ξ* of DE with different pre-stretch ratios at temperature *T* = 320 K, *T* = 340 K and 360 K are analyzed, as shown in Fig. 13. In this case, the pre-stretch *λ*_*P*1_ = 2.0, 3.0, 4.0 and 5.0. The results show that the non-elastic deformation decreases with the improve of the temperature at the same pre-stretch ratio. For the same temperature, the non-elastic deformation decreases with the rise of the pre-stretch ratio.

**Figure 14:**
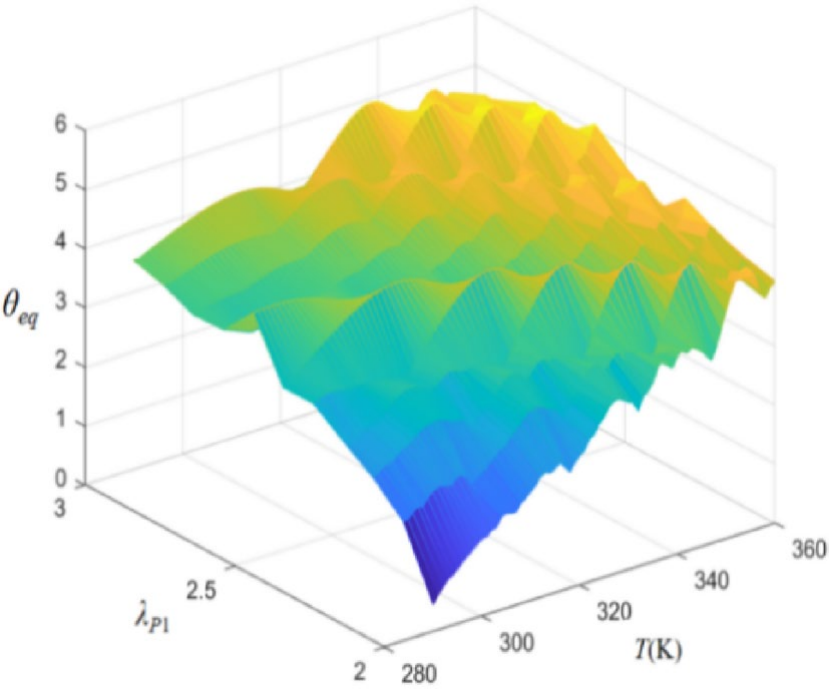
Equilibrium bending angle of DEMES for different values of pre-stretch ratios and temperatures.

Figure 14 shows the combined effects of temperature (*T*) and pre-stretch (*λ*_*P*1_) on the equilibrium bending angle of DEMES (*θ*_*eq*_). The results show that as the temperature increases, the equilibrium bending angle of DEMES increases. This is because the Young’s modulus of both the DE membrane and the flexible frame decreases as the temperature rises, resulting in a reduction in the overall stiffness of DEMES. In addition, the equilibrium bending angle of DEMES also increases with the increase of pre-stretch ratio. This is because the increase in pre-stretch stress can lead to an increase in the equilibrium bending angle of DEMES. The trend of the 3D rendering intuitively reflects the influence of temperature and pre-stretch ratio on DEMES deformation, that is, temperature and pre-stretch ratio promote the increase of DEMES bending angle.

### Analysis of the coupling effect of pre-stretch and temperature on DEMES under electric driving conditions

#### Analysis of the coupling effect of pre-stretch and temperature on DEMES under DC voltage driving conditions

**Figure 15:**
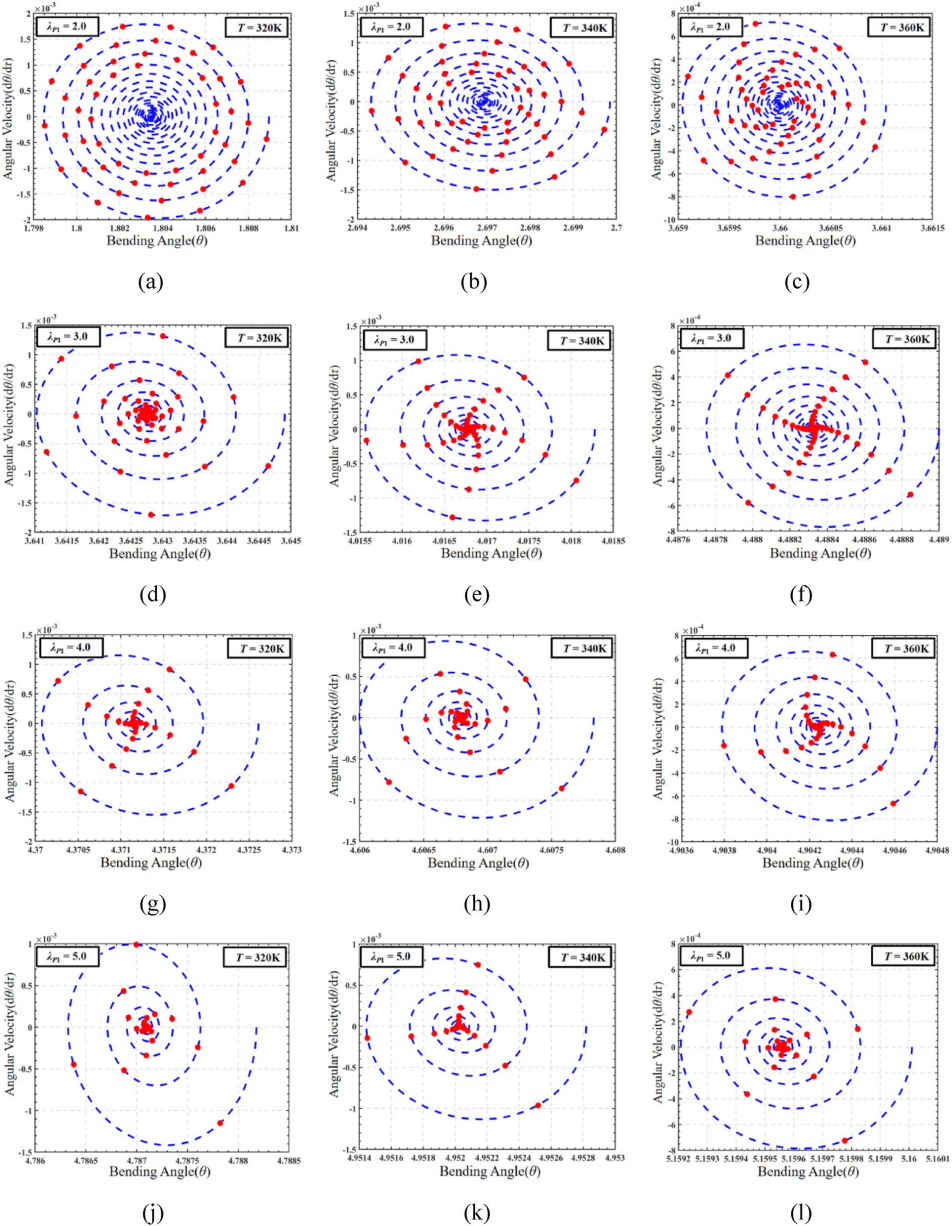
The phase paths and Poincaré maps of DEMES with different pre-stretch ratios and temperatures under DC driving conditions. (**a**), (**b**) and (**c**) *λ*_*P*1_ = 2.0, (**d**), (**e**) and (**f**) *λ*_*P*1_ = 3.0, (**g**), (**h**) and (**i**) *λ*_*P*1_ = 4.0, (**j**), (**k**) and (**l**) *λ*_*P*1_ = 5.0.

The influence of temperature cannot be ignored in the working process of DEMES, and the coupling effect of temperature and pre-stretch ratio also has great research value. In order to evaluate the coupling effect of pre-stretch and temperature on DEMES under DC voltage driving conditions, the phase paths and the corresponding Poincaré maps of the dynamic response of DEMES with different pre-stretch ratios at temperature *T* = 320 K, 340 K and 360 K are analyzed, as shown in Fig. 15. In this case, the pre-stretch *λ*_*P*1_ = 2.0, 3.0, 4.0 and 5.0. The initial conditions should be set to the equilibrium state results of Sect. “Analysis of the effect of pre-stretch on DEMES under voltage-free driving conditions”. In order to better study the effects of pre-stretch and temperature on the dynamic characteristics of DEMES under DC excitation, a DC voltage of 5000 V is selected to be applied to the DE menbrane without any other external excitation interference.

At a pre-stretch ratio of 2.0 and a temperature of 320 K, the phase path of DEMES shows uniform oscillations around the equilibrium angle, and these oscillations decay over time. The Poincaré map of DEMES shows significant divergence and ultimately exhibits a certain form of sustained oscillation, indicating that it has experienced non-periodic oscillations, as shown in Fig. 15a. When the temperature is 340 K, compared to the temperature of 320 K, the Poincaré phase map shows a decrease in the final sustained oscillation degree, as shown in Fig. 15b. When the temperature is 360 K, the Poincaré map of DEMES shows star divergence, indicating that DEMES is also in a non-periodic oscillation state, as shown in Fig. 15c. When the pre-stretch ratio increases to 3.0, the maximum angular velocity of DEMES significantly decreases under the temperature of 320 K, 340 K, and 360 K, resulting in weaker oscillation and faster decay rate of the system, as shown in Fig. 15d, e, and f. When the pre-stretch ratios are 4.0 and 5.0, the oscillation of DEMES further attenuates and eventually disappears, and the angular velocity decreases to zero, as shown in Fig. 15g–l. Therefore, under DC excitation, the pre-stretch ratio significantly affects the process of DEMES from the initial equilibrium state at room temperature to establishing a new equilibrium state. When the pre-stretch ratio *λ*_*P*1_ = 2, the pre-stretch stress inside the membrane is low, and the deformation process of DEMES is mainly driven by voltage, oscillating around the equilibrium position. On the contrary, when the prestretch ratio *λ*_*P*1_ ≥ 3, When the pre-stretch ratio is higher, the pre-stretch stress inside the membrane increases, and the DEMES is more obviously affected by the pre-stretch stress, at which time the voltage driving force effect is weakened, resulting in weakened oscillations. Finally, when the pre-stretch stress reaches a certain threshold, the oscillation decays to zero during the deformation process, and the Poincaré map takes the form of a center point. The research results indicate that under the excitation of DC voltage, DEMES is almost in a non-periodic oscillation state at various pre-stretch ratios, so the oscillation state of DEMES under DC excitation is uncontrollable. The next section will investigate the dynamic characteristics of DEMES under AC excitation.

### Analysis of the coupling effect of pre-stretch and temperature on DEMES under AC voltage driving conditions

When analyzing coupling effect of pre-stretch and temperature on the dynamic characteristics of DEMES under AC driving conditions, the initial conditions should be set to the equilibrium state results of Sect. “Analysis of the effect of pre-stretch on DEMES under voltage-free driving conditions”. The applied AC voltage can be given as Eq. (27). In order to better study the effects of pre-stretch and temperature on the dynamic characteristics of DEMES under AC excitation, a AC amplitude voltage of 5000 V is selected to be applied to the DE membrane without any other external excitation interference.

**Figure 16:**
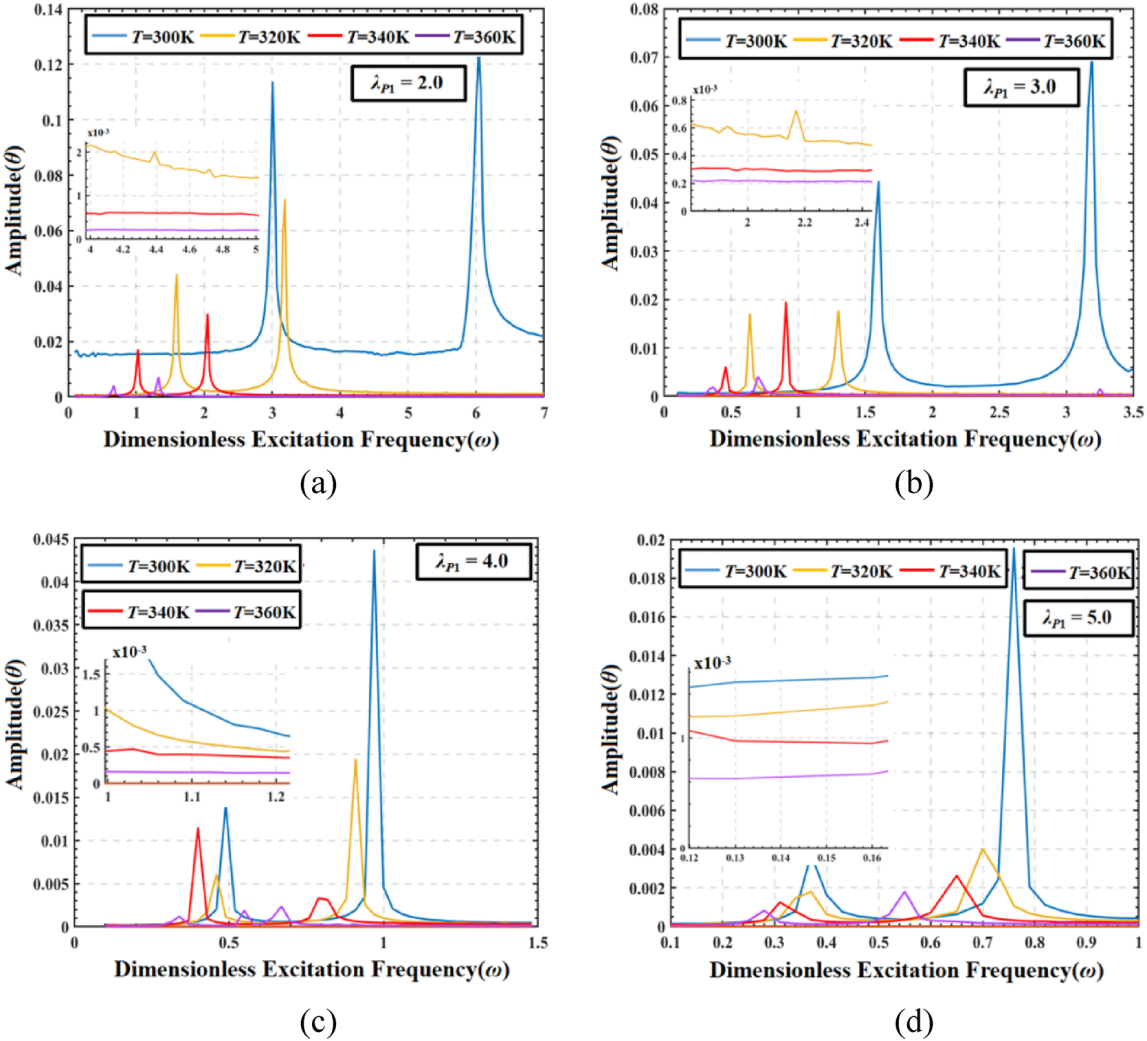
Amplitude frequency response of DEMES with different pre-stretch ratios and temperatures under AC driving conditions. (**a**) *λ*_*P*1_ = 2.0, (**b**) *λ*_*P*1_ = 3.0, (**c**) *λ*_*P*1_ = 4.0 and (**d**) *λ*_*P*1_ = 5.0.

Figure 16 shows the amplitude frequency response curves of the DEMES with different pre-stretch ratios (*λ*_*P*1_ = 2.0, 3.0, 4.0 and 5.0) at different temperatures (*T* = 300 K, 320 K, 340 K and 360 K). In addition, a small window is used in each image to display the amplitude variation of DEMES at different temperatures under different stages of dimensionless excitation frequencies.

The results indicate that when the pre-stretch ratio is constant, the resonant frequency (maximum amplitude frequency curve) and oscillation amplitude decrease with increasing temperature. This is mainly due to the increase in temperature leading to a decrease in the overall stiffness of DEMES. In addition, the results also show that when the temperature is in constant, the resonant frequency and oscillation amplitude reduced with the improve of pre-stretch ratio. This may be due to the increase in pre-stretch ratio, which leads to an increase in pre-stretch stress inside the DE membrane, and a smaller resonance frequency is required to trigger the resonance phenomenon.

**Figure 17:**
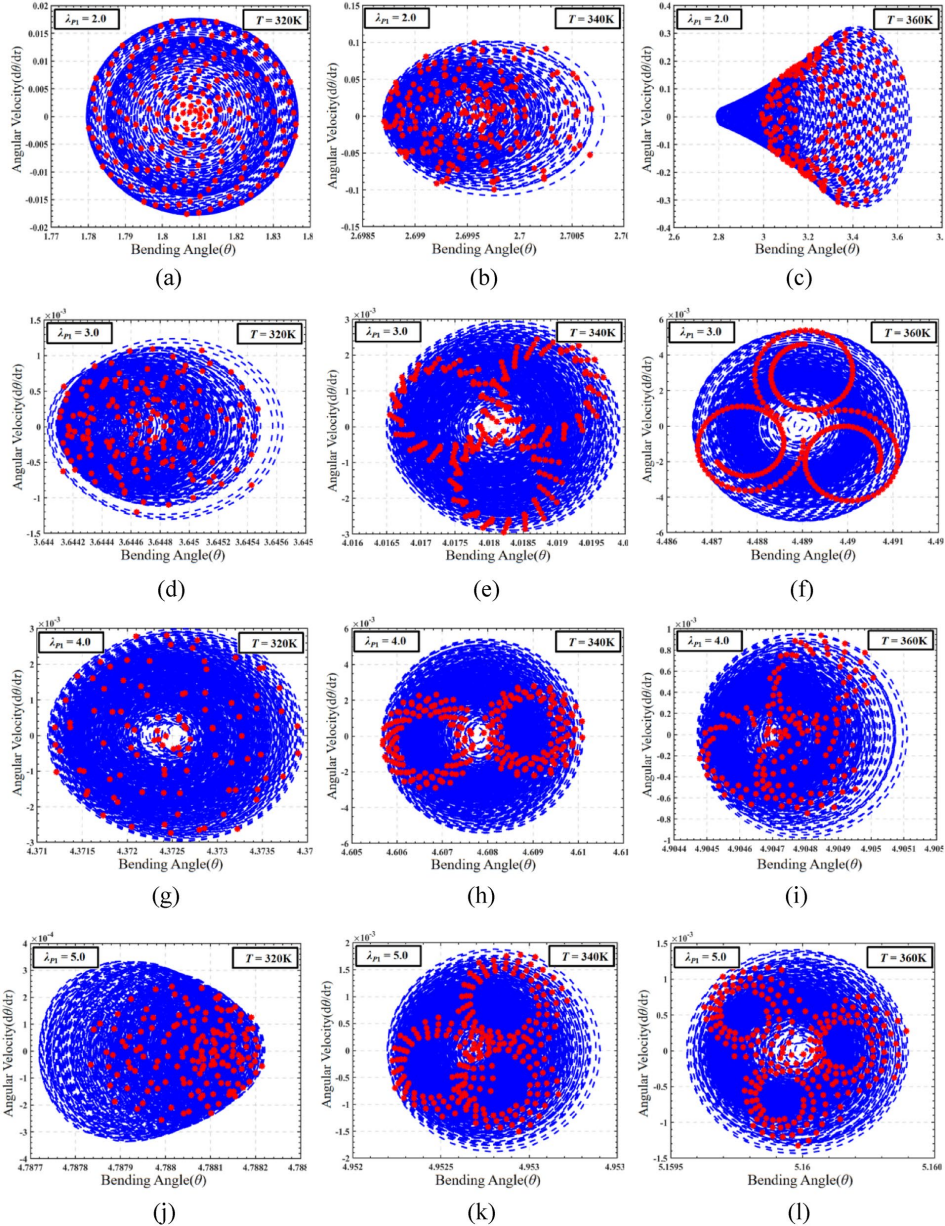
AC analysis under the coupling influence of DEMES pre-stretch load and temperature load. (**a**), (**b**) and (**c**) *λ*_*P*1_ = 2.0, (**d**), (**e**) and (**f**) *λ*_*P*1_ = 3.0, (**g**), (**h**) and (**i**)* λ*_*P*1_ = 4.0, (**j**), (**k**) and (**l**) *λ*_*P*1_ = 5.0.

In order to study the effects of pre-stretch and temperature on the dynamic characteristics of DEMES, the phase path and Poincaré map of the system under AC load are plotted, as shown in Fig. 17. The selected temperature ranges are 320 K, 340 K, and 360 K, with an AC voltage amplitude of 5000 V. The frequency used is determined to be the average resonance frequency of 320 K, 340 K, and 360 K at various pre-stretch ratios discussed in the previous chapters, which is 1.5. Under the conditions of a pre-stretch ratio of 2.0 and a temperature load of 320 K, the phase path of DEMES exhibits uniform oscillation near the equilibrium angle, and the amplitude gradually decreases with time. The Poincaré map depicts a uniform divergent distribution around the center point of the equilibrium angle with certain distribution regularity, indicating the non-periodic oscillation state of the DEMES system, as shown in Fig. 17a. At a temperature of 340 K, the Poincaré map exhibits significant divergence and weakened distribution patterns compared to 320 K, as shown in Fig. 17b. At a temperature of 360 K, the phase path shows a uniform attenuation trend towards the final equilibrium angle, and during the fluctuation process, the overall oscillation form ultimately stabilizes at the equilibrium angle. Compared with the temperature of 320 K, the Poincaré map shows uneven divergence, which means it is in non-periodic oscillation, as shown in Fig. 17c. As the pre-stretch ratio increases, under a temperature load of 320 K, the Poincaré map shows an irregular and dense distribution of divergent patterns, indicating the non-periodic oscillation form of DEMES in this state, as shown in Fig. 17d, g, and j. At a temperature load of 340 K, the Poincaré map of DEMES changed from an uneven divergent distribution at 320 K to circular distributions, indicating that the current DEMES system is in a transitional state, as shown in Fig. 17e, h and k. Finally, under a temperature load of 360 K, the Poincaré map of DEMES shows three different closed-loop states, which are further evolved from the circular distribution at 340 K. In summary, when in a relatively low temperature state, the Poincaré maps of DEMES exhibit irregular divergence at various pre-stretch ratios, indicating that the structure of DEMES is in a non-periodic oscillation state at relatively low temperatures. When in a relatively high temperature state, the Poincaré maps of DEMES begin to transition from a divergent distribution to a local circular distribution. Moreover, when the DEMES is in a relatively high temperature state, as the pre-stretch ratio increases, the Poincaré map shows a trend from local closed-loop to local divergence, and finally returns to local closed-loop. This indicates that when under relatively high temperature conditions, the DEMES structure undergoes a transition from a periodic oscillation state to a non-periodic oscillation state, and ultimately recovers to a quasi periodic oscillation state due to the increase in pre-stretch stress. The research results indicate that compared to DC excitation, the dynamic characteristics of DEMES under AC excitation are more diverse, which means that the working stability of DEMES under AC excitation is controllable. A lower pre-stretch ratio and lower temperature are unfavorable for the working state of DEMES. Choosing an appropriate higher pre-stretch ratio can significantly improve the dynamic performance of DEMES, such as 2.5–3.0. An excessively high pre-stretch ratio may cause unstable strong oscillations.

## Conclusion

An analytical dynamic model for DEMES considering the coupling effect of pre-stretch and temperature factors is presented in this paper. The results can be summarized as follows. Under voltage-free driving conditions, the final equilibrium angle of the DEMES increases when the pre-stretch ratio is increased. The deformation process of DEMES is more significantly affected by pre-stretch under DC voltage loading. Under AC voltage loading, the DEMES phase diagram undergoes a transition from uniform oscillations to non-uniform oscillations and then to periodic oscillations as the pre-stretch ratio increases, indicating that the DEMES undergoes a transition from periodic to non-periodic motion in the process of moving from a low pre-stretch ratio to a high pre-stretch ratio. The results also show that the temperature has a significant impact on the resonant frequency of DEMES system. Under the same pre-stretch ratio, as the temperature increases, the resonance frequency of the DEMES system shifts towards lower values, while the resonance amplitude decreases. Under low pre-stretch ratio and low temperature conditions, DEMES exhibited non-periodic oscillation. When in a high temperature state, with the increase of pre-stretch stress, DEMES exhibits a process from periodic oscillation state to non-periodic oscillation state, and finally returns to quasi-periodic oscillation state. This paper indicates that the pre-stretch and temperature has a significant impact on the dynamic performance of DEMES. The bending angle, non-elastic deformation, resonant frequency, and stability of DEMES can be jointly adjusted by pre-stretch ratio and ambient temperature. The research results can provide theoretical guidance for the dynamic performance and stability evolution of DEMES.

## Expectation

This paper theoretically analyzes the effects of temperature and pre-stretch factors on the equilibrium state, DC and AC response of DEMES from the perspective of dynamic modeling. The conclusion shows that pre-stretch and temperature have a significant impact on the dynamic performance of DEMES. The bending angle, non elastic deformation, resonant frequency, and stability of DEMES can be adjusted by the ratio of temperature to pre-stretch.

From the perspective of dynamic modeling for DEMES actuators, this paper establishes a dynamic control equation for DEMES using the neo-Hookean hyperelastic model. The hyperelastic model is a model used to describe the nonlinear mechanical behavior of materials over a large strain range. The hyperelastic model includes: a homogeneous isotropic hyperelastic model assuming the material has isotropy, an anisotropic hyperelastic model considering the material’s anisotropy, an electromechanical coupling model considering the influence of electric field on material properties, a thermo mechanical coupling model considering the influence of temperature on material properties, and so on. This paper focuses on the isotropy of DE thin membranes. In order to further investigate the influence of DE thin membrane anisotropy on the dynamic response of DEMES actuators, an analytical framework can be established to analyze the impact of DE thin membrane anisotropy on the dynamic response of DEMES actuators. Relevant studies have shown that anisotropic DEMES actuators require less time to reach equilibrium than isotropic DEMES actuators, and theoretically have better dynamic performance. As the anisotropy parameter of the DE membrane increases, the equilibrium angle obtained by the DEMES structure increases, indicating that the anisotropy of the DE membrane has a significant impact on the initial state of the structure. This indicates that the anisotropy of DE thin membranes has important research value on the impact of DEMES actuators, and there is increasing scientific interest in endowing DE materials with anisotropy to improve their driving performance. Developing anisotropic DEMES actuators with good usability is of great research significance.

In addition to the influence of environmental temperature and the pre-stretch ratio of DE membrane on the dynamic performance stability of DEMES, environmental humidity, environmental magnetism, and the magnitude of excitation voltage may also have an impact on the working performance of DEMES. From the actual working environment of DEMES, the influence of environmental humidity cannot be ignored. In order to study the influence of environmental humidity on the actuation characteristics of DEMES, in the future, the influence of environmental humidity on the dielectric constant, elastic modulus, and viscous damping of DEMES can be introduced, combined with the standard linear solid model and Euler Lagrange dynamic equation, to construct a dynamic control equation for DEMES (including DE membrane and flexible frame) affected by humidity. Through numerical simulation, study and analyze the influence of humidity on the stiffness, bending deformation, and dynamic response evolution of DEMES under DC/AC excitation. By combining factors such as environmental temperature, humidity, pre-stretch state of DE membrane, and viscoelasticity of DE membrane, the analysis of the actuation characteristics of DEMES will be more accurate, and the effect on improving the actuation ability of DEMES will be more significant.

## Data Availability

All data generated or analysed during this study are included in this published article (and its Supplementary Information files). If someone wants to request the data from this study, please contact the corresponding author: Yanmin.zhou@tongji.edu.cn.
